# Chickpeas’ and Lentils’ Soaking and Cooking Wastewaters Repurposed for Growing Lactic Acid Bacteria

**DOI:** 10.3390/foods12122324

**Published:** 2023-06-09

**Authors:** Gonçalo Nuno Martins, Angela Daniela Carboni, Ayelén Amelia Hugo, Paula Cristina Castilho, Andrea Gómez-Zavaglia

**Affiliations:** 1 CQM—Centro de Química da Madeira, Universidade da Madeira, Campus da Penteada, 9020-105 Funchal, Portugal; goncalo.martins@staff.uma.pt (G.N.M.); pcastilho@staff.uma.pt (P.C.C.); 2 Center for Research and Development in Food Cryotechnology (CIDCA, CCT-CONICET La Plata), La Plata RA1900, Argentinaahugo@biol.unlp.edu.ar (A.A.H.)

**Keywords:** galacto-oligosaccharides, pulses, *Lactiplantibacillus plantarum*, circular economy, waste management, culture medium

## Abstract

Legumes processing involves large amounts of water to remove anti-nutrients, reduce uncomfortable effects, and improve organoleptic characteristics. This procedure generates waste and high levels of environmental pollution. This work aims to evaluate the galacto-oligosaccharide (GOS) and general carbohydrate composition of legume wastewaters and assess their potential for growing lactic acid bacteria. Legume wastewater extracts were produced by soaking and/or cooking the dry seeds of chickpeas and lentils in distilled water and analysed using high-performance liquid chromatography with refractive index detection. GOS were present in all extracts, which was also confirmed by Fourier transform infrared spectroscopy (FTIR). C-BW extract, produced by cooking chickpeas without soaking, provided the highest extraction yield of 3% (g/100 g dry seeds). Lentil extracts were the richest source of GOS with degree of polymerization ≥ 5 (0.4%). *Lactiplantibacillus plantarum* CIDCA 83114 was able to grow in de Man, Rogosa, and Sharpe (MRS) broth prepared by replacing the glucose naturally present in the medium with chickpeas’ and lentils’ extracts. Bacteria were able to consume the mono and disaccharides present in the media with extracts, as demonstrated by HPLC and FTIR. These results provide support for the revalorisation of chickpeas’ and lentils’ wastewater, being also a sustainable way to purify GOS by removing mono and disaccharides from the mixtures.

## 1. Introduction

Pulses are an important component of the human diet and contain proteins, oligosaccharides, dietary fibre, minerals, and antioxidant polyphenols [1]. Although their consumption has important health benefits (e.g., the reduction of cholesterol and the prevention of the development of diabetes and cancer [1,2]), certain innocuous but uncomfortable associated effects (e.g., flatulence) can make their ingestion undesirable. Therefore, before consumption, soaking and cooking treatments are used to reduce these effects, as well as to enhance the bioavailability of important compounds and improve organoleptic characteristics, such as texture and flavour [3].

According to the latest definition, prebiotics are substrates selectively utilised by the host microorganisms conferring a health benefit [4]. Pulses contain various types of oligosaccharides with prebiotic effects, including galacto-oligosaccharides (GOS) and fructo-oligosaccharides (FOS), that are not absorbed or hydrolysed in the upper part of the gastrointestinal tract. GOS are composed of a varying number of galactose (Gal) units and a terminal glucose (Glu) or sucrose residue, resulting in different degrees of polymerization (DP). The type of GOS present in pulses, specifically α-GOS, belong to the raffinose-family of oligosaccharides (RFO) and are responsible for causing flatulence. Raw pulses are considered high in GOS, with values ranging from 1 to 10% [5]. Soaking and cooking treatments help to reduce the content of α-GOS, as they are partially leached and degraded by enzymatic action [6].

The processing of legumes, such as chickpeas and lentils, requires the use of large amounts of water, resulting in the generation of waste and high levels of environmental pollution [7]. Reusing industrial by-products or discards is becoming increasingly important to improve the sustainability of food production and reduce waste. This approach is aligned with the principles of the circular economy, an economic system based on business models that prioritize reducing, reusing, recycling, and recovering materials in the production, distribution and consumption processes, instead of the traditional ‘end-of-life’ concept [8]. Although the utilization of food industry waste is increasingly applied to the food itself, the water from the processing of these foods continues to be majorly discarded. Aquafaba (commonly employed as an egg substitute) is one of the most well-known examples of reusing water from the treatment of chickpeas. Some of the studied applications of legumes’ wastewaters include the production of baked goods, ice-cream, and pastry products [9,10]. Colucci Cante et al. [11] evaluated the fermentation of bean blanching wastewaters as a way to valorise them. 

Lactic acid bacteria have played a crucial role in the production of fermented products for centuries. They are capable of fermenting various substrates, producing lactic acid and other metabolites with health-promoting and technological properties—such as reducing spoilage microorganisms, acidification—and enhancing sensory attributes [12]. These microorganisms have been successfully used in the production of lactose-free dairy foods and beverages through lactose hydrolysis, such as bread and other cereal-based foods, and alcoholic beverages, such as wine [13]. Recently, a review of the use of lactic acid bacteria in the production of traditional food products from Latin America through the fermentation of raw materials, such as tubers, cereals, and fish, was published [12]. Lactic acid fermentation was also studied as a way to improve the nutritional and sensorial aspects of legume and fruit beverages, with the finding that fermentation not only was useful to enhance these characteristics, but also to extend the shelf life of products [14,15]. Lactic acid bacteria have a GRAS status (“generally recognized as safe”) from the FDA (USA), and as such, they are increasingly being used both in fermentation processes and as functional ingredients [16].

The objective of this work was to evaluate the carbohydrate composition, particularly the GOS, of wastewaters produced during the soaking and cooking of chickpeas and lentils, and their potential as carbohydrate sources for the growth of *Lactiplantibacillus plantarum* CIDCA 83114, a lactic acid bacteria strain isolated from kefir grains. This strain was selected because of to its resistance to preservation processes (including drying treatments), its high stability during storage, its potential probiotic activity, and its ease of growing on simple media [17,18]. The wastewaters were recovered from food producing industries and this work aims to valorise these resources in a circular economy mind-set.

## 2. Materials and Methods

### 2.1. Materials

Chickpea (*Cicer arietinum* L.) (Continente, Matosinhos, Portugal) and lentil (Lens culinaris Medikus var. variabilis) (Don Elio, Santa Fe, Argentina) seeds were acquired at local supermarkets. Vivinal^®^ GOS syrup was kindly provided by Friesland Campina Ingredients (Veghel, The Netherlands). The microbiology medium de Man, Rogosa, and Sharpe (MRS) broth was purchased at Sigma-Aldrich^®^ (Burlington, MA, USA). Other reagents used were acquired from common vendors.

### 2.2. Preparation of Legumes’ Treatment Wastewater Samples

**Figure 1:**
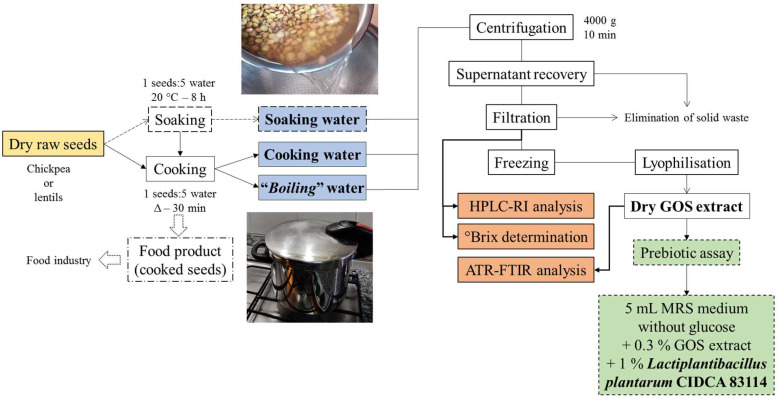
Experimental procedures for the production and characterisation of chickpea- and lentil-derived extracts and the bacterial growth-potential assessment assay using *Lactiplantibacillus plantarum* CIDCA 83114.

GOS-containing extracts (wastewaters) from chickpea and lentils were obtained following a similar method to that described by Han and Baik [3]. Three treatments were carried out: soaking, soaking and cooking, and cooking without soaking of the seeds (Figure 1). A 1:5 (*w*/*v*, seeds: distilled water) ratio was used for the three treatments in both legumes.

Raw dried seeds were soaked for 8 h at 20 °C. After that, the seeds were strained, obtaining the waters from the soaking chickpeas (C-SW) and lentils (L-SW). The soaked chickpeas were then cooked in a pressure cooker (C-CW) and the soaked lentils in a pot (L-CW), both for 30 min. Thirdly, extracts were obtained by boiling dry (*i.e.*, without soaking) chickpeas (C-BW) and lentils (L-BW) for 30 min in a pressure cooker and a pot, respectively.

After the thermal treatments, the water extracts were cooled to 20–25 °C and centrifuged (15 min, 4000× *g*) (Heraeus Instruments, Hanau, Germany). The supernatants were filtered using filter funnels with a porosity of 4 (10–16 µm pores). Afterwards, the samples were freeze-dried in a Martin Christ Gefriertrocknungsanlagen GmbH freeze-dryer (Alpha 1–2 LD Plus, Osterode, Germany).

### 2.3. HPLC-RI Analysis

The extracts obtained in Section 2.2 were filtered with 0.45 µm of cellulose acetate filters (Frilabo, Maia, Portugal), and the carbohydrates were determined using high-performance liquid chromatography with refractive index (HPLC-RI) detection (UltiMate 3000, Dionex, Sunnyvale, CA, USA). A ReproGel-Na column (Dr. Maisch, Ammerbuch, Germany) of 250 × 8 mm and a particle size of 9 μm was used with a CARBOSep CHO 411 pre-column (Concise Separations, San Jose, CA, USA) at 80 °C. The RI detector (Shodex RI-101) was maintained at 50 °C. Degasified filtered ultrapure water was used as mobile phase with a flow rate of 500 µL/min. The obtained chromatograms were analysed with Chromeleon 6.80 software (Dionex Corporation, Sunnyvale, CA, USA).

Because of the absence of GOS standards in the market, different concentrations of Vivinal^®^ GOS syrup were used as standards, to determine the retention times of the sugars of interest and to calibrate the analytical method used. The syrup was composed of Gal, Glu, lactose (Lac), and short chain β-GOS (up to DP = 7) as shown in Table 1 (Friesland Campina DOMO, Amersfoort, The Netherlands, 2017) [19], alongside the retention times determined for each carbohydrate species. A calibration curve for fructose (Fru), whose retention time is 11.4 min, was obtained from a previous work [20]. The HPLC-RI calibration is detailed in Supplementary Material S1. From the determined calibration curves, the sugar composition of the samples was expressed as g/100 g of fresh extract.

### 2.4. Extraction Yield

The extraction yields were calculated based on the HPLC-RI analysis and the concentration (g/100 g of fresh extract) determined for each carbohydrate detected. The sugar content on a dry basis (g/100 g of dry extracts) and their extraction yield (g/100 g of dry seeds) were calculated by Equations (1) and (2), respectively, after performing °Brix measurements (RX-100, Atago digital refractometer) and determining the volume of fresh extract obtained. The yields for GOS-DP ≥ 5, GOS-DP = 4, GOS-DP = 3, Lac, Glu, Gal, and Fru’s extraction were calculated separately from each calibration curve.

(1) $$\text{Sugar content in}\;g/100\;g\;\text{dry extract}=\frac{{{\text{Sugar}}_{(g/100\;g\;\text{fresh extract})}}_{}}{{{}^{\circ}Brix}_{\left(g/100\;g\;\text{fresh extract}\right)}}\times 100$$

(2) $$Yield\;in\;g/100\;g\;dry\;seeds=\frac{{Sugar\;}_{(g/L)}\times V_{extract(L)}}{m_{dry\;seeds\;(g)}}\times 100$$

### 2.5. FTIR Analysis

The freeze-dried samples obtained in Section 2.2 were analysed by a Fourier transform infrared spectrometer (Spectrum Two, Perkin Elmer, Waltham, MA, USA) with attenuated total reflectance (ATR) equipment with a diamond crystal (UATR Two, Perkin Elmer, Waltham, MA, USA). The spectra were registered in the 4000–400 cm^−1^ range by co-adding 32 scans with 4 cm^−1^ spectral resolution at 20 °C. The spectra were analysed using spectrum software (Perkin Elmer, Waltham, MA, USA). The Vivinal^®^ GOS syrup was also analysed as a reference material for a complex carbohydrate mixture.

### 2.6. Microbiological Assays with Oligosaccharide Mixtures

*Lactiplantibacillus plantarum* CIDCA 83114 was isolated from kefir grains [21] and maintained frozen at −80 °C in 120 g/L of non-fat milk solids. Cultures were grown in an MRS broth [22] at 37 °C overnight in aerobic conditions to obtain approximately 10^10^−10^11^ of CFU/mL (stationary phase). Then, they were harvested by centrifugation at 10,000× *g* for 10 min at 4 °C, and the pellets were washed twice with a phosphate buffered saline (PBS) solution (K_2_HPO_4_ 0.144 g/L; NaCl 9.00 g/L; Na_2_HPO_4_ 0.795 g/L, pH 7) and used to evaluate the microbial growth potential of the water extracts derived from chickpeas and lentils (Section 2.2).

To that aim, the microorganisms were inoculated (1% *v*/*v*) in MRS (5 mL) broth formulated without glucose (composition in Supplementary Material S2) and supplemented at 0.3% *w*/*v*, either with the extracts obtained in Section 2.2 or with Gal, Glu, Lac, or Vivinal^®^ GOS syrup. All the solutions were sterilized by filtration (0.45 μm pore filter diameter, Frilabo, Maia, Portugal). Blank controls were carried out by inoculating the strain in MRS without the addition of extracts or sugars. The samples were incubated at 37 °C for 24 h and then serially diluted in PBS, plated on MRS agar, and incubated at 37 °C for 48 h in aerobic conditions. The results were expressed as colony-forming units (CFU) per millilitre (CFU/mL). To calculate the growth potential of the samples, the CFU/mL values for the samples were referred to those of the blank control (MRS without sugars) (Equation (3)).

(3) $$log\;CFU/mL=log\left({CFU/mL}_{Sugar}-{CFU/mL}_{Blank}\right)$$

The consumption of carbohydrates during fermentation was evaluated by HPLC-RI and FTIR, following similar protocols as those explained in Section 2.3 and Section 2.5.

Figure 1 shows a summary of the experimental procedures. 

A final microbiological experiment was performed by growing *L. plantarum* CIDCA 83114 at 37 °C for 24 h in a C-BW extract prepared in distilled water at 51 g/L (MRS concentration). After growing, the culture was centrifuged and the supernatant was filtered and analysed in the HPLC-RI for comparison with the initial sample. The FTIR spectra were also recorded for the supernatant after lyophilization and compared with the C-BW extract’s spectrum obtained in Section 2.5.

### 2.7. Statistical Analysis

The obtained results were evaluated by one-way analysis of variance (ANOVA), using InfoStat software. When this analysis expressed statistical differences (*p* < 0.05), intragroup comparisons were tested using the Tukey test.

## 3. Results and Discussion 

### 3.1. HPLC-RI Results and Extraction Yield

**Figure 2:**
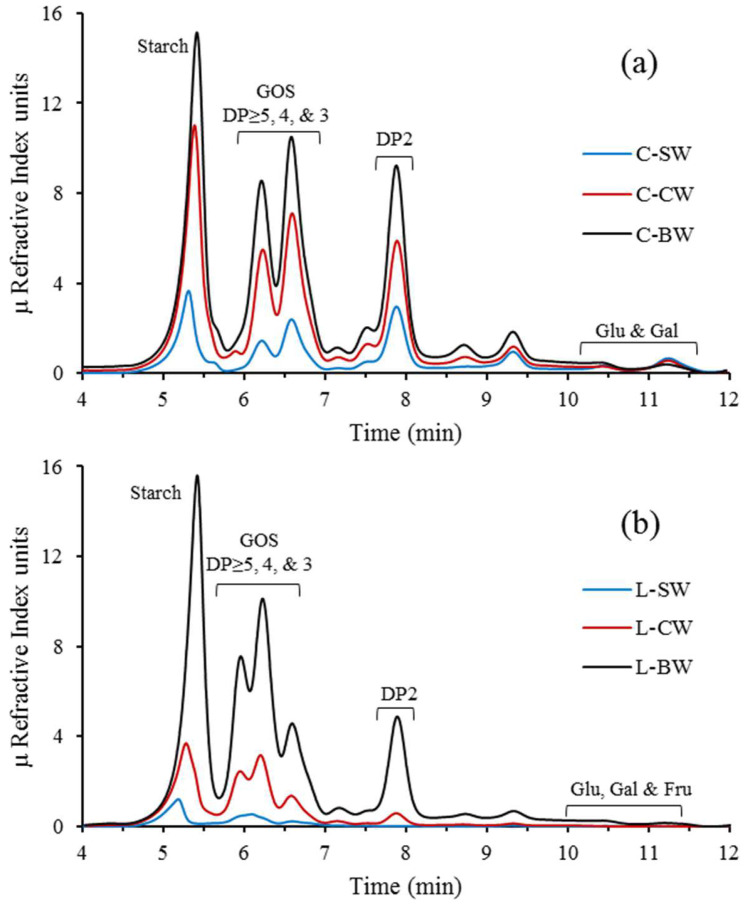
HPLC-RI chromatograms of soaking (SW), cooking (CW), and cooking without soaking “boiling” (BW) waters from (**a**) chickpeas and (**b**) lentils.

The chromatograms obtained from the fresh extracts of chickpeas and lentils exhibited similar features, especially in the boiling water extracts (Figure 2) and resembled those of Vivinal^®^ GOS syrup (S1). Considering that the same seeds-to-water weight proportion was used in all procedures, the results indicate that chickpeas are a richer source of saccharides than lentils, because the chromatograms showcase a higher number of peaks, and those in common have higher intensities. GOS were found in higher quantities in the chickpea extracts, and lentils provided much fewer mono- and disaccharides. Fru was only detected in the lentil extracts; however, the juxtaposition of its peak (11.4 min) with that of Gal (11.2 min) may cause its eclipsing in chickpea waters. In all the chromatograms peaks found between 7 and 8 min and at around 9 min have not yet been identified. The prominent peak present in all samples at around 5 min can be attributed to a Glu-composed polysaccharide, most likely starch. This inference is supported by the observation of starch in the presence of iodine during an assay conducted on the legumes’ wastewaters from the present study. The chromatograms’ resemblance to that of Vivinal (S1) highlights the relevance of its use for HPLC-RI calibration, in view of the absence of GOS standards in the market. 

Table 2, Table 3 and Table 4 show the GOS, DP2 sugars, Glu, Gal, and Fru contents in g/100 g of fresh and dry extract and the extraction yield in g/100 g of dry seeds used for the soaking and cooking, respectively. Fresh extract represents the extract after the centrifugation and filtration steps, which is useful to analyse the sample for direct utilization. Dry extract is expressed on a dry basis and facilitates the manipulation and storage steps. 

When evaluating the soaking and cooking treatments, soaking provided the lowest extraction yields, but the obtained mixtures were purer, since the measured ºBrix values (total dissolved solids) of the fresh extracts were closer to the calculated total quantifiable sugar content. C-BW was the fresh extract with the highest total GOS, total mono- and disaccharides, and total saccharides, when compared with the rest of treatments and with lentil extracts. The soaking methods are effortless, and all the extracted material is highly hydrophilic and water soluble. Contrarily, the cooking (C-CW) and boiling (C-BW and L-BW) processes enable the extraction of compounds with lower solubility [9], resulting in a significantly lower concentration of total GOS in the dry extracts (Table 3). It is worth noting that the combined contents of GOS-DP ≥ 5 and DP = 4 are the same for all chickpea extracts (16 g/100 g of dry extract); however, C-SW possesses almost 10% more GOS-DP = 3, twice the amount of DP2 sugars, and thrice the amount of Glu and Gal than C-CW and C-BW. This trend was not observed for the lentil-derived extracts, with the total saccharide content being approximately 36–40% in all the dry extracts. L-CW was the lentil extract with the lowest total mono- and disaccharides; however, Fru was detected but was not quantified because its area was below the limit of quantification. According to the USDA [23], raw lentil possesses up to 0.27 g of Fru/100 g, and L-BW extraction produced 0.04 g of Fru/100 g of lentils; for chickpea, no Fru values are reported by the USDA [23], and no extraction of this sugar was observed. When comparing the dried extracts of both legumes, it can be noted that the total GOS, total mono- and disaccharides, and total saccharides content were significantly lower in lentils than in chickpeas.

According to the USDA [23], raw lentil possesses 0.27 g of Fru/100 g, and L-BW extraction produced 0.04 g of Fru/100 g of lentils, while no values are reported for chickpea.

In terms of the total GOS production, as seen in Table 4, the C-BW was the most interesting extract, with a 3% extraction yield (*i.e.*, 3 g of the total GOS (of which half are DP = 3 sugars) were produced out of 100 g of dry chickpea seeds used), and without the need for soaking the legume. Similarly, the L-BW extract stands out as the most efficient source for GOS-DP ≥ 5’s extraction, specifically, producing 0.4 g out of 100 g of dry legume seeds. This results in less utilization of resources and generates fewer waste products. In terms of total mono- and disaccharides, chickpeas’ extract obtained significantly higher values than lentils’. This is also true for the total GOS content, except for L-BW. 

Serventi [24] evaluated the cooking water of legumes (after soaking), finding that this water possesses high amounts of oligosaccharides. In the present study, it was observed that treatments that involve the use of heat (cooking and boiling) led to a higher extraction of GOS than soaking, which can be correlated with the results of Liu and Serventi [25], who showed that the process of cooking after soaking can lead to a loss of 60–85% of oligosaccharides in legumes, compared to soaking (50–75%). 

**Table 2:** Concentration of different sugars as g/100 g of fresh extract obtained from chickpeas and lentils *.

Compound (%)	Chickpea Extracts			Lentil Extracts		
	C-SW	C-CW	C-BW	L-SW	L-CW	L-BW
GOS-DP ≥ 5	0.01 ± 0.00	0.03 ± 0.00	0.04 ± 0.00	0.03 ± 0.00	0.08 ± 0.00	0.22 ± 0.00
GOS-DP = 4	0.07 ± 0.00	0.26 ± 0.00	0.39 ± 0.01	0.02 ± 0.00	0.15 ± 0.00	0.49 ± 0.01
GOS-DP = 3	0.15 ± 0.00	0.41 ± 0.00	0.59 ± 0.01	0.03 ± 0.00	0.10 ± 0.00	0.29 ± 0.01
Lac/DP = 2	0.17 ± 0.00	0.33 ± 0.01	0.50 ± 0.01	0.01 ± 0.00	0.04 ± 0.00	0.28 ± 0.01
Glu	0.05 ± 0.00	0.04 ± 0.00	0.07 ± 0.00	0.02 ± 0.00	0.02 ± 0.00	0.05 ± 0.00
Gal	0.05 ± 0.00	0.05 ± 0.00	0.04 ± 0.00	0.00 ± 0.00	0.00 ± 0.00	0.02 ± 0.00
Fru	N.D.	N.D.	N.D.	N.D.	<L.O.Q.	0.02 ± 0.01
Total GOS	0.23 ± 0.00 ^b^	0.69 ± 0.01 ^d^	1.02 ± 0.01 ^f^	0.09 ± 0.00 ^a^	0.33 ± 0.01 ^c^	0.99 ± 0.01 ^e^
Total mono- + disaccharides	0.26 ± 0.01 ^c^	0.42 ± 0.01 ^e^	0.61 ± 0.02 ^f^	0.04 ± 0.00 ^a^	0.06 ± 0.00 ^b^	0.37 ± 0.02 ^d^
Total saccharides ^†^	0.49 ± 0.01 ^c^	1.11 ± 0.01 ^d^	1.64 ± 0.05 ^f^	0.12 ± 0.00 ^a^	0.39 ± 0.01 ^b^	1.36 ± 0.04 ^e^
° Brix (total solids)	0.5	1.8	2.8	0.3	1.0	3.7

DP = degree of polymerization; SW = soaking water; CW = cooking water; BW = boiling water; N.D. = not detected; <L.O.Q. = below the limit of quantification. ^†^ Total saccharides = total quantifiable saccharides. * Results are expressed as average ± SD. Different letters in the same row indicate significant differences among samples (*p* < 0.05); n = 3.

**Table 3:** Concentration of different sugars as g/100 g of dried extracts obtained from chickpeas and lentils *.

Compound (%)	Chickpea Extracts			Lentil Extracts		
	C-SW	C-CW	C-BW	L-SW	L-CW	L-BW
GOS-DP ≥ 5	1.62 ± 0.13	1.53 ± 0.02	1.59 ± 0.13	10.56 ± 0.30	7.92 ± 0.40	5.95 ± 0.10
GOS-DP = 4	14.73 ± 0.07	14.24 ± 0.22	14.00 ± 0.37	7.15 ± 0.26	15.29 ± 0.35	13.13 ± 0.34
GOS-DP = 3	29.68 ± 0.06	22.70 ± 0.07	21.10 ± 0.47	10.99 ± 0.47	9.86 ± 0.04	7.73 ± 0.18
Lac/DP = 2	33.39 ± 0.35	18.32 ± 0.29	17.97 ± 0.52	3.88 ± 0.12	4.25 ± 0.24	7.49 ± 0.23
Glu	9.66 ± 0.55	2.34 ± 0.14	2.35 ± 0.10	7.35 ± 0.19	2.12 ± 0.09	1.47 ± 0.11
Gal	9.54 ± 0.27	2.58 ± 0.07	1.60 ± 0.05	0.44 ± 0.07	0.31 ± 0.01	0.42 ± 0.05
Fru	N.D.	N.D.	N.D.	N.D.	<L.O.Q.	0.66 ± 0.20
Total GOS	46.02 ± 0.26 ^f^	38.47 ± 0.31 ^e^	36.68 ± 0.98 ^d^	28.69 ± 1.03 ^b^	33.07 ± 0.79 ^c^	26.80 ± 0.61 ^a^
Total mono- + disaccharides	53.25 ± 0.22 ^f^	23.24 ± 0.50 ^e^	21.91 ± 0.67 ^d^	11.67 ± 0.38 ^c^	6.68 ± 0.34 ^a^	10.04 ± 0.59 ^b^
Total saccharides ^†^	98.61 ± 1.43 ^e^	61.71 ± 0.81 ^d^	58.60 ± 1.65 ^c^	40.36 ± 1.41 ^b^	39.75 ± 1.14 ^b^	36.84 ± 1.20 ^a^

DP = degree of polymerization; SW = soaking water; CW = cooking water; BW = boiling water; N.D. = not detected; <L.O.Q. = below the limit of quantification. ^†^ Total saccharides = total quantifiable saccharides. * Results are expressed as average ± SD. Different letters in the same row indicate significant differences among samples (*p* < 0.05); n = 3.

**Table 4:** Chickpeas’ and lentils’ extraction yields expressed as g of sugar/100 g of dried seeds *.

Compound (%)	Chickpea Extracts			Lentil Extracts		
	C-SW	C-CW	C-BW	L-SW	L-CW	L-BW
GOS-DP ≥ 5	0.03 ± 0.00	0.10 ± 0.00	0.14 ± 0.01	0.12 ± 0.00	0.25 ± 0.01	0.35 ± 0.01
GOS-DP = 4	0.29 ± 0.00	0.92 ± 0.01	1.21 ± 0.03	0.08 ± 0.00	0.48 ± 0.01	0.78 ± 0.02
GOS-DP = 3	0.58 ± 0.00	1.47 ± 0.00	1.83 ± 0.04	0.13 ± 0.01	0.31 ± 0.00	0.46 ± 0.01
Lac/DP = 2	0.65 ± 0.01	1.19 ± 0.02	1.56 ± 0.04	0.05 ± 0.00	0.13 ± 0.01	0.44 ± 0.01
Glu	0.19 ± 0.01	0.15 ± 0.01	0.20 ± 0.01	0.09 ± 0.00	0.07 ± 0.00	0.09 ± 0.01
Gal	0.19 ± 0.01	0.17 ± 0.00	0.14 ± 0.00	0.01 ± 0.00	0.01 ± 0.00	0.02 ± 0.00
Fru	N.D.	N.D.	N.D.	N.D.	<L.O.Q.	0.04 ± 0.01
Total GOS	0.90 ± 0.00 ^b^	2.49 ± 0.02 ^e^	3.15 ± 0.01 ^f^	0.34 ± 0.01 ^a^	1.05 ± 0.03 ^c^	1.59 ± 0.04 ^d^
Total mono- + disaccharides	1.04 ± 0.00 ^d^	1.49 ± 0.00 ^e^	1.87 ± 0.00 ^f^	0.14 ± 0.00 ^a^	0.21 ± 0.01 ^b^	0.59 ± 0.03 ^c^
Total saccharides ^†^	1.92 ± 0.03 ^c^	4.00 ± 0.05 ^e^	5.03 ± 0.01 ^f^	0.47 ± 0.02 ^a^	1.26 ± 0.04 ^b^	2.18 ± 0.07 ^d^

DP = degree of polymerization; SW = soaking water; CW = cooking water; BW = boiling water; N.D. = not detected; <L.O.Q. = below the limit of quantification. ^†^ Total saccharides = total quantifiable saccharides. * Results are expressed as average ± SD. Different letters in the same row indicate significant differences among samples (*p* < 0.05); n = 3.

### 3.2. FTIR

**Figure 3:**
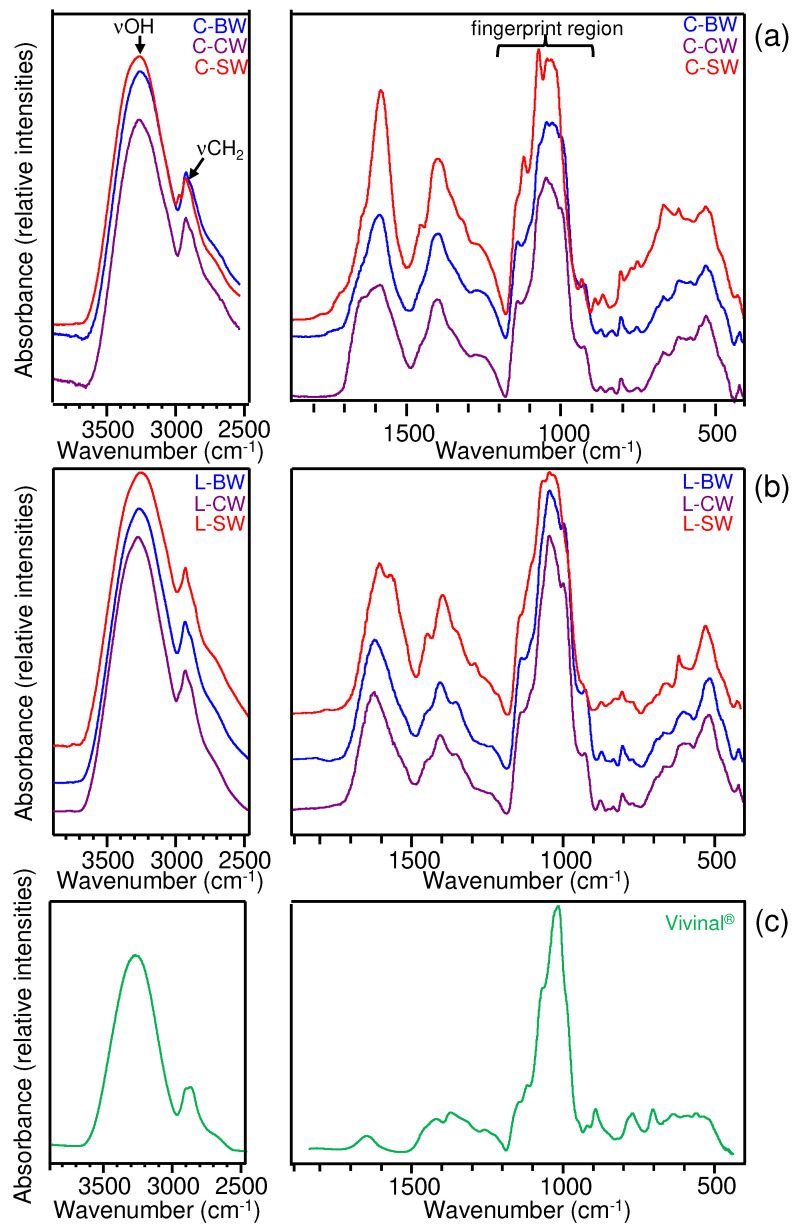
FTIR spectra of the chickpeas’ soaking (C−SW) and cooking (C−CW and “boiling” C−BW) waters (**a**); of the lentils’ equivalent extracts (L−SW, L−CW, and L−BW) (**b**); and of the Vivinal^®^ GOS syrup (**c**). νOH and νCH_2_ denote the stretching vibrational modes of the OH and CH_2_ groups.

Figure 3 shows the FTIR spectra obtained for the dried chickpeas’ and lentils’ extracts, and for the reference material, Vivinal^®^ GOS syrup. The spectra of all the extracts were very similar and superimposable. This indicates that the soaking process has no noteworthy qualitative effect on the biomolecules detected by a spectral analysis, meaning that both soaking and cooking processes enable the extraction of similar compounds. The spectrum obtained for the Vivinal^®^ GOS syrup, which corresponds to a mixture of β-GOS, Lac, Glu, and Gal, was also similar to those of the extracts. The bands shared between the samples and the Vivinal^®^ GOS syrup included the broad one at 3500–3000 cm^−1^ (OH stretching) and those in the fingerprint region (1200–800 cm^−1^), arising from the C-O-C glycosidic linkage, the COH bending, and the C-C stretching vibrational modes, that collectively provide a characteristic pattern for each carbohydrate [26,27]. The differences observed between the Vivinal^®^ GOS syrup and the extracts in this region may be related to the fact that the former does not contain polysaccharides. As stated before, we hypothesize that the peak at 5 min observed in the HPLC chromatograms of the extracts corresponds to starch. This conclusion is further supported by the FTIR analysis. Romano et al. [28] also observed starch-related bands in the 1250–800 cm^−1^ region when evaluating the FTIR spectra of quinoa flour. The main differences between the samples’ and the Vivinal^®^ GOS syrup’s spectra were related to the relative intensities of the bands observed in the still-undiscussed double-bond stretching and local symmetry regions, around 1800–1500 cm^−1^ and 1500–1200 cm^−1^, respectively. The bands detected in the former can be attributed to the presence of unsaturated bonds (e.g., in the C=O groups found in carbohydrates) and unspecific CH_2_ bending vibrations, whereas those found in the latter can be ascribed to vibrations arising from C-O groups, also observed in carbohydrates [29]. 

### 3.3. Microbiological Assay with Oligosaccharide Mixtures

**Figure 4:**
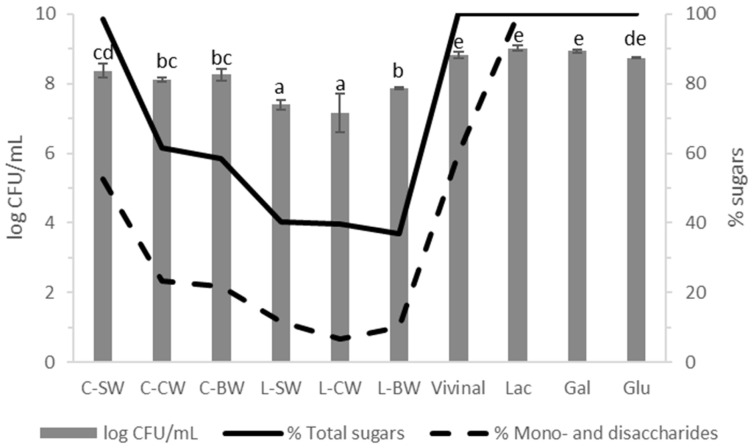
Results of the growth-potential assessment assay in log CFU/mL (left axis) and the samples’ initial sugar composition (right axis) for the chickpea and lentil extracts, and for Vivinal, lactose (Lac), galactose (Gal), and glucose (Glu). Results are expressed as average ± SD. Different letters indicate significant differences among samples (*p* < 0.05); n = 4.

Figure 4 shows the results of the 24 h bacterial growth assays obtained for all the extracts, the standards, and the Vivinal^®^ GOS syrup used as a reference material. All the sample extracts were capable of promoting the growth of *L. plantarum* CIDCA 83114, showcasing a growth potential comparable to that of the standard sugars assayed. The chickpea extracts were the most successful in this regard, leading to bacterial counts close to those obtained for Glu (the sugar present in the standard MRS medium composition) and for Vivinal^®^ GOS syrup, which is composed of β-GOS, unlike α-GOS present in the extracts. L-BW was the best extract from the lentils’ counterpart.

The joint content of mono- and disaccharides does not explain the observed results, where extracts such as C-BW—which only presents 20% of these sugars—showcase bacterial counts close to those obtained for Glu (100%), at 8.2 and 8.7 log CFU/mL, respectively. This indicates that other sugars present, namely GOS, are being utilized by the microorganisms. However, when comparing the total sugar content (total quantifiable sugars) of C-BW (60%) with that of the Vivinal^®^ GOS syrup (100%) this correlation falls short. There are two possible explanations. The first is that *L. plantarum* CIDCA 83114 is capable of more efficiently utilising α-GOS (such as those present in the extracts), than β-GOS (such as those found in the Vivinal^®^ GOS syrup) for its growth, which has been previously reported for some probiotic cultures. Oh et al. [30] studied the growth effect of different α- and β-GOS on several non-probiotic and probiotic bacterial strains and determined that the oligosaccharide structure influences their growth, even amongst different strains of the same species. The second, and most likely reason, is that the unknown polysaccharide found in the extracts (Figure 2) is also being metabolized by *L. plantarum* CIDCA 83114. 

**Figure 5:**
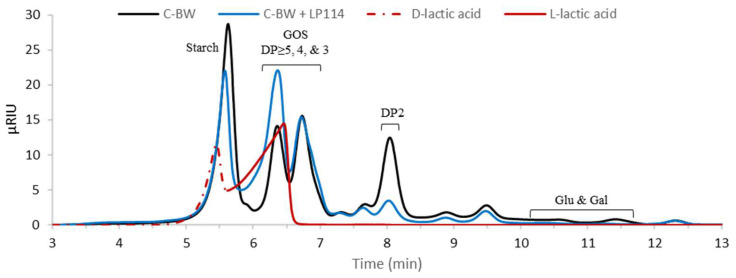
HPLC-RI chromatograms of C-BW before and after the growth of *L. plantarum* CIDCA 83114 (LP114) in water for 24 h at 37 °C. A chromatogram obtained for a racemic mixture of D- and L-lactic acid is also shown.

To answer this question, *L. plantarum* CIDCA 83114 was grown at 37 °C for 24 h in C-BW (51 g/L) and the final medium was analysed in the HPLC-RI (Figure 5). It was observed that Glu (10.5 min) and Gal (11.4 min) peaks entirely disappeared, while that of DP = 2 sugars (8 min) greatly decreased its intensity after fermentation. The intensity of GOS peaks at around 6–7 min was not altered. The increase in the GOS-DP = 4 peak (6.3 min) can be explained considering the formation of L-lactic acid, whose retention time is similar to that of GOS-DP = 4. After fermentation, the C-BW extract’s chromatogram had 97% of the total area of the initial C-BW sample before fermentation, and a 51 g/L C-BW sample had 18.7 g/L total GOS. As GOS were unaffected by the bacterial metabolic activity, they are expected to still be present at this concentration after fermentation. Interestingly, the polysaccharide peak at around 5 min decreased its intensity. Therefore, it can be concluded that *L. plantarum* CIDCA 83114 preferably utilises sugars where Glu is present. As previously mentioned, the polysaccharide is very likely starch, a molecule composed of Glu units, whereas GOS—in particular RFO—at most contain only one Glu residue. This explains the values of CFU/mL for extracts such as C-CW, C-BW, and L-BW (Figure 4), that showed low concentrations of mono- and disaccharides (as a whole) but whose chromatograms (Figure 2) exhibited a very prominent polysaccharide peak. Future investigations will delve further into this matter.

**Figure 6:**
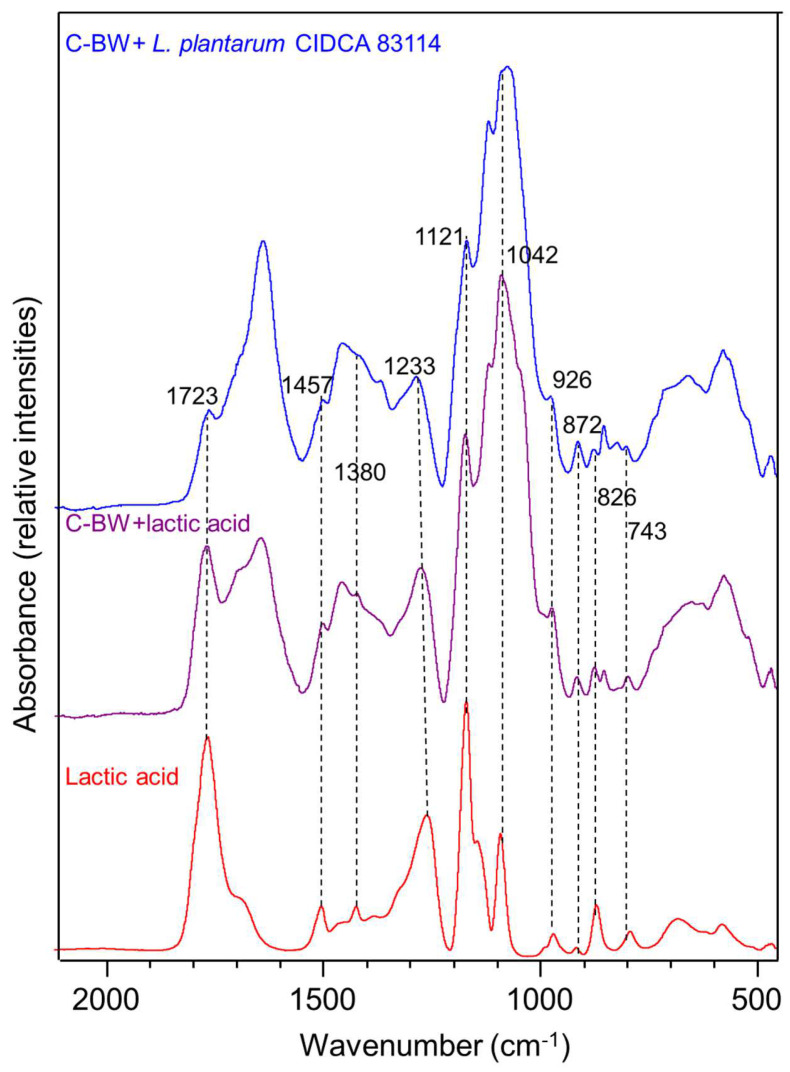
FTIR spectra of C−BW after fermentation with *L. plantarum* CIDCA 83114 (**top**), of C−BW spiked with lactic acid (**middle**), and of lactic acid (**bottom**).

After fermentation, the C-BW sample (sans the bacteria) was freeze-dried and analysed using FTIR (Figure 6, top). Although the spectrum is pretty much similar to that of the initial C-BW extract (Figure 3), it exhibited some new bands, which are denoted in Figure 6. The most noticeable change was the shoulder occurring at 1723 cm^−1^, probably arising from a C=O stretching vibrational mode. Such a functional group is possibly due to the formation of L-lactic acid, as previously identified in the HPLC-RI analysis (Figure 5). The presence of L-lactic acid was further confirmed by spiking the initial C-BW extract powder with a small amount of L-lactic acid and analysing the mixture using FTIR (Figure 6, middle spectrum). As expected, an increase in some bands (corresponding to L-lactic acid) was observed and such bands corresponded to those new bands previously observed for C-BW fermented with *L. plantarum* CIDCA 83114. These bands were definitely confirmed as belonging to L-lactic acid by analysing the spectrum of the pure compound (Figure 6, bottom), which resembled that found in the National Institute of Standard and Technology database [31]. 

These results show that legume wastewaters, such as C-BW alone, have the nutritional requirements for growing *L. plantarum* CIDCA 83114, and GOS content does not change according to the fermentation process for this strain and GOS are still available at the end of the process. The fermentation of legume extracts with bacterial strains can therefore be an efficient purification method to remove mono-and disaccharides from α-GOS, before their employment in other industries and applications. If legume extracts were to be incorporated in food products, the functionality of legume extracts could be improved by the addition of probiotic bacteria with beneficial effects for human health. 

## 4. Conclusions

Legumes are a rich source of prebiotic compounds, such as GOS. Utilising the wastewaters produced during the soaking and cooking of chickpeas and lentils proved to be a cost-effective and efficient method for their extraction and recovery. Cooking chickpeas provided the highest GOS extraction yields, while lentils’ cooking waters were the richest source of GOS-DP ≥ 5 compounds, with low concentrations of mono- and disaccharides. Microbial experiments carried out with *L. plantarum* CIDCA 83114 showed that legume wastewaters can be valorised and effectively repurposed as microbiological growth media for lactic acid bacteria, leading to less waste disposal. Considering that this strain consumed the monosaccharides and greatly diminished the DP = 2 carbohydrates present in the C-BW extract, its use could be further explored as a potential purification method for GOS mixtures.

The obtained results allow for the elucidation of the initial aspects of the use of wastewaters from the processing of legumes, in order to avoid environmental contamination and obtain compounds with prebiotic activity, mainly at the laboratory level. The simplicity of this approach and its ease of scalability make it highly suitable for future implementation by industrial stakeholders. 

## Figures and Tables

**Table 1:** Content (%) and retention times of saccharides present in Vivinal^®^ GOS syrup [19].

	DP7 ^a^	DP6 ^a^	DP5 ^a^	DP4	DP3	Lac/DP2 ^b^	Glu	Gal	Total	Total GOS ^c^
**Content (%)**	7.4			10.8	22.0	37.4	21.1	1.3	100.0	40.2
**Retention** **time (min)**	5.5–5.9			6.4	7.0	8.1	10.4	11.2		

^a^ GOS species with DP = 7, 6, and 5 were treated as one due to poor resolution of the obtained peaks. ^b^ Lac present in Vivinal^®^ GOS syrup was used as a reference for other similar DP = 2 carbohydrates (*i.e.*, disaccharides) with the same retention time. ^c^ Total GOS indicates the sum of GOS DP = 3, DP = 4, DP = 5, DP = 6, and DP = 7′s contents.

## Data Availability

The data presented in this study are available on request from the corresponding author.

## Supplementary Materials

The following supporting information can be downloaded at: https://www.mdpi.com/article/10.3390/foods12122324/s1.
