# Supplementary material for: Chickpeas’ and Lentils’ Soaking and Cooking Wastewaters Repurposed for Growing Lactic Acid Bacteria
Source: Foods. 2023 Jun 9;12(12):2324. doi: 10.3390/foods12122324 (PMC10297098; doi:10.3390/foods12122324)
Supplement: Supplementary file 1 [file foods-12-02324-s001.zip › 10_Supplementary Material S1.pdf]

Supplementary Material S1

Composition of Vivinal® GOS syrup and HPLC-RI chromatogram, experimental conditions and the retention times, linearity, limit of detection (LOD), limit of quantification (LOQ), and intra- and inter-day precision parameters determined for the HPLC-RI analysis of different carbohydrate species.

A. Composition of Vivinal® GOS syrup as described by Friesland Campina DOMO (2017)[1]

	DP7[a]	DP6[a]	DP5[a]	DP4	DP3	Lac/DP2[b]	Glu	Gal	Total	Total GOS[c]
%		7.4		10.8	22.0	37.4	21.1	1.3	100.0	40.2

[a] GOS species with DP = 7, 6, and 5 were treated as one GOS species.

[b] Lac present in Vivinal® GOS syrup was used in this work as a reference for other similar DP = 2 carbohydrates (*i.e.*, disaccharides) with the same retention time.

[c] Total GOS indicate the sum of DP = 3 + DP = 4 + DP = 5 + DP = 6 + DP = 7 GOS.

B. Calibration of HPLC-RI method for the quantification of carbohydrates using solutions of Vivinal® GOS syrup at different concentrations

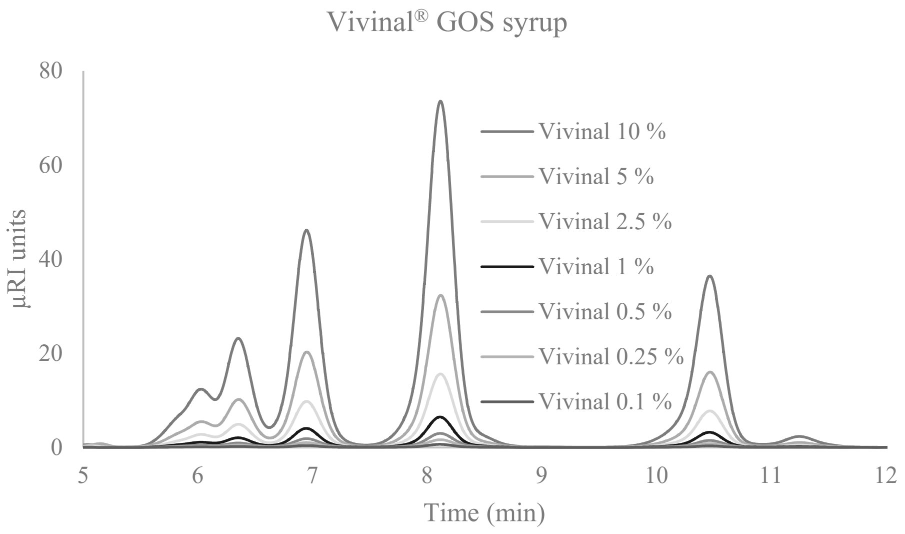

[1] Vivinal® GOS PT. A submission to the UK Food Standards Agency requesting consideration of Substantial Equivalence in accordance with Regulation (EC) No 258/97 concerning novel foods and novel food ingredients. (Non-Confidential Version). Available at: https://acnfp.food.gov.uk/sites/default/files/gos.pdf. Accessed: 13th May 2022.

Carbohydrate species		GOS-DP ≥ 5[a]	GOS-DP = 4	GOS-DP = 3	Lactose/DP = 2[b]	Glucose	Galactose
**Chromatographic run**	**Solvent**	100 % degasified filtered ultrapure water					
	**Time (min)**	20					
	**Temperature (°C)**	80					
**Injection volume (μL)**		5					
**RI detector's temperature (°C)**		50					
**Retention time (min)**		5.9-6.2	6.2-6.5	6.9-7.1	7.9-8.1	10.4	11.2
**Linearity[c]**	**m**	8.3	5.9	5.8	5.6	5.3	6.8
	**b**	0.0	-0.1	-0.1	-0.1	-0.0	0.0
	**R^2**	0.9987	0.9976	0.9976	0.9488	0.9977	0.9995
	**Δ [ ], % (w/w)[c1]**	0.007-0.736	0.011-1.076	0.022-2.202	0.037-3.742	0.021-2.112	0.001-0.131
**LOD[d]**	**3.3*SD/m (% [w/w])**	0.0005					
**LOQ[e]**	**10*SD/m (% [w/w])**	0.0015					
**Precision[f] (% RSD[f1])**	**Intra-day[f2]**	8.8	8.8	8.8	8.8	8.8	8.8
	**Inter-day[f3]**	14.4	14.6	14.6	14.3	14.4	15.0

[a] Galacto-oligosaccharides (GOS) with degree of polymerization (DP) ≥ 5 were treated as one compound due to poor chromatographic resolution between these species' peaks.

[b] Calibration for lactose present in Vivinal® GOS syrup was used as a reference for other similar DP = 2 carbohydrates (disaccharides) with the same retention time.

[c] Calibration curves determined for each carbohydrate species present in Vivinal® GOS syrup. [c1] Δ [ ] = concentration range in % (w/w) of each calibration curve.

[d] LOD (Limit of Detection) and [e] LOQ (Limit of Quantification) were calculated from the calibration curve of galactose by the "standard deviation of the response and the slope" method, in which *m* is the slope of the calibration curve and *SD* is the standard deviation of the area of the lowest point in the curve. The value calculated for galactose was chosen as representative for all carbohydrates since it is the sugar with the lowest concentration assayed.

[f] Precision parameters were determined by the analysis of solutions of 1 and 10 % of Vivinal® GOS syrup (n = 6) and calculating the % of the [f1] RSD (Relative Standard Deviation) of the areas obtained for each analysis in the same day ([f2] intra-day) and in three non-consecutive days ([f3] inter-day) of analysis.

C. Correlation of the areas determined for glucose present in the Vivinal® GOS syrup solutions analysed and in solutions prepared using standard glucose (Riedel-de Haën) at the same concentrations found in the Vivinal® GOS syrup solutions

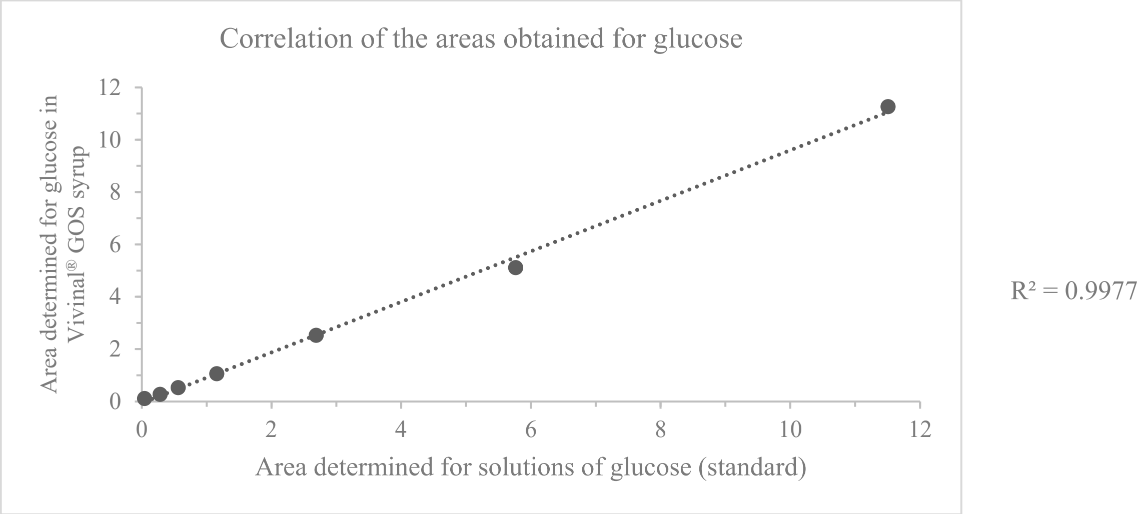

		**Concentrations of**						
**Vivinal® GOS syrup solutions**	% (w/w)	0.1	0.25	0.5	1	2.5	5	10
**Glucose in Vivinal® GOS syrup solutions**	% (w/w)	0.02	0.05	0.11	0.21	0.53	1.06	2.11
	g/L	0.2	0.5	1.1	2.1	5.3	10.6	21.1
**Std glucose solutions**	g/L	0.2	0.5	1.1	2.1	5.3	10.6	21.1

Std: standard.

D. A calibration curve for fructose (Merck) was determined as y = 1.4× + 0.0 (R^2 = 0.9990). Its retention time was attributed to be 11.4 min (Figueira, 2020)[2]

[2] Figueira, O. A. da S. (2020). Profile analysis of oligosaccharides in yacon (*Smallanthus sonchifolius*) roots - extraction optimization and inulin hydrolysis [Universidade da Madeira]. http://hdl.handle.net/10400.13/3143
