# Supplementary material for: Chickpeas’ and Lentils’ Soaking and Cooking Wastewaters Repurposed for Growing Lactic Acid Bacteria
Source: Foods. 2023 Jun 9;12(12):2324. doi: 10.3390/foods12122324 (PMC10297098; doi:10.3390/foods12122324)
Supplement: Supplementary file 1 [file foods-12-02324-s001.zip › 11_Supplementary Material S2.pdf]

Supplementary Material S2

Composition of the de Man, Rogosa, and Sharpe medium prepared without glucose.

Component	Concentration	Brand
Peptone	20 g/L	Biokar (France)
Yeast extract	5 g/L	Oxoid (United Kingdom)
Dipotassium hydrogen phosphate	2 g/L	J. T. Baker (USA)
Sodium acetate trihydrate	5 g/L	Anedra (Argentina)
Triammonium citrate	2 g/L	Mallinckrodt (USA)
Magnesium sulphate heptahydrate	0.2 g/L	ICN Biomedicals (USA)
Manganese sulphate tetrahydrate	0.05 g/L	Sigma (USA)
Tween 80	1 mL for 1 L	Biopack (Argentina)
